# Coming back home to start up a business? A comparison between youth from rural and urban backgrounds in China

**DOI:** 10.3389/fpsyg.2022.962419

**Published:** 2022-08-09

**Authors:** Chih-Hung Yuan, Dajiang Wang, Lihua Hong, Yehui Zou, Jiayu Wen

**Affiliations:** ^1^School of Economics and Commerce, University of Electronic Science and Technology of China, Zhongshan Institute, Zhongshan, China; ^2^College of Entrepreneurship, Wenzhou University of Technology, Wenzhou, China; ^3^School of Marxism, University of Electronic Science and Technology of China, Zhongshan Institute, Zhongshan, China; ^4^School of Art and Design, University of Electronic Science and Technology of China, Zhongshan Institute, Zhongshan, China

**Keywords:** young people, return-home entrepreneurship, barriers to entrepreneurship, support mechanism, rural revitalization

## Abstract

Youth entrepreneurship is regarded as an important part of rural revitalization. Against the backdrop of the rural revitalization strategy, the Chinese government has introduced many policies to encourage return-home entrepreneurship among young people. However, highly educated youth have a lower willingness to return home for entrepreneurship, and prefer urban entrepreneurship or getting a job in a city. Therefore, this study used a two-stage approach to explore the factors that influence young people’s contribution to the development of their homeland, the barriers they face, and the support mechanisms they need. The study found that many barriers affect young people’s intention to return home for entrepreneurship. In rural areas, young people consider lagging environmental development to be the biggest barrier. In urban areas, infrastructure, lack of entrepreneurial experience, and funding are the factors that concern young people the most. As they have limited entrepreneurial experience, young people in both rural and urban areas have a high demand for shared entrepreneurial experience, as well as entrepreneurship courses and mentoring. The government and universities should remove the barriers faced by young people, provide more assistance, improve the environment for young people engaging in return-home entrepreneurship, and form a good entrepreneurial ecology.

## Introduction

Youth entrepreneurship has long been regarded as an important part of revitalization due to the potential of young people to make special contributions to job opportunities, communities, and economic prospects (Geldhof et al., 2014; Dias et al., 2019). In particular, the rural areas, suburban areas, and underperforming areas that suffer from the problems of depopulation and insufficient infrastructure and those that rely heavily on agricultural activities can benefit from the economic diversification brought about by entrepreneurial activities (Sá et al., 2019). In the past, rural areas were defined by economic activities related to specific resources (especially agriculture and livestock farming), open spaces (such as farmland and mountains), as well as socio-spatial characteristics (such as population density and distance from major cities). Newer approaches put more emphasis on realistic social representation when defining rural areas, highlighting how people pursue rural ideals and strive to achieve them in their daily lives (Labrianidis, 2006). Therefore, new opportunities have emerged in rural areas, including increased demand for entertainment and rural facilities, as well as higher-quality rural products and services, such as tourism (Pato and Teixeira, 2016; Duan et al., 2020; Saridakis et al., 2021).

Scholars have discussed youth rural entrepreneurship from the points of view of intention (Bouichou et al., 2021), barriers (Senou and Manda, 2022), motives (Bednaříková et al., 2016), support mechanisms (Adeyanju et al., 2021; Shi et al., 2021), entrepreneurial ability (Ataei et al., 2020), mentoring (Shittu, 2017), and social networks (Singh Sandhu et al., 2011). For example, Senou and Manda (2022) discovered the impact of access to finance on rural youth entrepreneurship, and pointed out that age, education, poverty status, experience, working in the agricultural sector, and the existence of a bank branch are important determinants of rural youth’s access to finance. Ataei et al. (2020) explored the impact of entrepreneurial competencies on entrepreneurial intention of rural youth. The results revealed that from the most to the least important factors underpinning rural youth’s willingness to start SMEs were the strategic, communicative, psychological, and opportunistic competencies.

However, we found few studies on young people’s engagement in return-home entrepreneurship. There is a growing interest in urban entrepreneurship among academics, with a focus on high-growth technology industries. Rural entrepreneurship is a unique area of entrepreneurship research and practice, which offers alternative opportunities for local development that do not necessarily follow the mainstream literature (Fortunato, 2014). Second, there are still major research gaps in the basic information concerning how young people perceive their communities and their own potential to participate in rural economies (de Guzman et al., 2020). Young people have advantages in terms of their attitudes and motivations, which are reflected in their high sensitivity to new information and technologies and the rapid adjustment of expectations, compared with older people. They are more willing to accept new ideas and continue to have strong curiosity and ability to learn new things, new knowledge, and new concepts. Young people can translate what they hear, see, and think through their innovative minds and practical ability and effectively support ongoing entrepreneurial activities by providing new ideas and a fresh impetus for rural economic development. Finally, few existing papers concurrently discuss the barriers to young people’s engagement in return-home entrepreneurship and the support mechanisms they need, especially in China.

To increase young people’s intention to return home for entrepreneurship and create jobs, the Chinese government launched three flagship programs, namely “Mass Entrepreneurship and Innovation,” the “Rise of Central China Plan (2016–2025),” and the “Rural Revitalization Strategy Plan (2018–2022).” There has been a wave of return-home entrepreneurship in China, which is essentially the return migration of more and more peasant workers from cities to their rural homes, who become self-employed afterward. According to a report on return-home entrepreneurship, the average age of return-home entrepreneurs in China is 46.6 years, and 71.6% of return-home entrepreneurs are 50 years old and below (Chen, 2018). Young and middle-aged people are the main source of return-home entrepreneurs. The same report revealed that the education level of the majority (57.0%) of return-home entrepreneurs is junior high school or below. Those who have graduated from high school account for 39.5%, and only 2.11% have a university degree.

The exodus of rural youth is often related to receiving higher education and entering the labor market (Bednaříková et al., 2016). In China, the number of university graduates has grown from 8.2 million in 2018 to 8.74 million in 2020, which is a record high. The proportion of university graduates choosing to work in new first-tier cities (including Beijing, Shanghai, Guangzhou, and Shenzhen Mycos Institute, 2020) rose from 48% in 2015 to 56% in 2019 (Mycos Institute, 2020). Given the decline of rural economies and the migration from rural areas, academics and policymakers are increasingly looking to entrepreneurship and local business development as a means of rural revitalization (de Guzman et al., 2020). Yu and Artz (2019) investigated alumni of a US university and found that individuals who grew up in rural areas were more likely to become rural entrepreneurs when compared to their peers who grew up in urban areas. Therefore, it is necessary to encourage highly educated youths to engage in return-home entrepreneurship in China.

With the call for more entrepreneurship and more entrepreneurial activities, it is important to understand the factors that influence return-home entrepreneurship (Sun et al., 2019). In this article, young people’s engagement in return-home entrepreneurship refers to young people choosing to return home and start new businesses after finishing their higher education studies away from their hometowns. As they are highly educated, they have advantages over local entrepreneurs, while having stronger adaptability compared with those from outside. Furthermore, most previous studies focused on either rural or urban residents, especially the youth, and some scholars suggested that greater attention should be paid to different aspects in different regions when formulating entrepreneurial policies (Freire-Gibb and Nielsen, 2014; Sun et al., 2019). There is still much potential in comparisons between rural and urban areas.

Research shows that employment growth is the main path of urban and rural economic development, while its main mechanism is entrepreneurship (Ataei et al., 2020). However, young people may be inexperienced and reluctant to take risks. Therefore, the purpose of this study was to use various methods to explore the factors that influence young people’s engagement in return-home entrepreneurship, including the barriers and support mechanisms. Framed as a multi-method study, we drew on a design to collect data using a two-stage approach to explore the opinions of the youth.

The contributions of this study are as follows: First, after an extensive literature review of youth entrepreneurship, this study found that most studies adopted a single research approach to observing the phenomenon of youth entrepreneurship. In this study, we employed a mixed approach that integrated quantitative and qualitative elements to investigate the experiences and preferences of the youth. Second, relevant empirical research is usually done on a maturity scale, and may not fit the situation in China. This study adopted a crowdsourcing survey to draft more appropriate questions according to the participants’ feedback, with consideration of the literature. Third, for studies on both agriculture and youth entrepreneurship, hometown factors are rarely taken into account, as usually only infrastructure construction (Tian et al., 2021). Based on the participants’ feedback, infrastructure construction was expanded to become hometown factors, including education, geographical location, economic development, and resources. Lastly, few studies have examined barriers and support mechanisms at the same time, and the literature on urban-rural comparisons is not plentiful.

## Methodology overview

As the Chinese government vigorously encourages return-home entrepreneurship among young people, there is an urgent need to increase our understanding of the related barriers and support mechanisms. This study employed a two-step approach to explore Chinese young people’s perceptions of the barriers to and assistance for return-home entrepreneurship. In this study, the following conditions were considered in relation to young people’s engagement in return-home entrepreneurship and the definitions thereof: (1) “Young people” refers to people aged 15–35 (Brixiová et al., 2015) and (2) “return-home entrepreneurship” refers to people, who have been trained or educated in higher education institutions away from home, having the intention to or having already returned to their hometown to start a new business. The following is an explanation of the development of the items and the questionnaire survey.

Given the different scenarios, there has not been much research on young people’s engagement in return-home entrepreneurship in China (Yu et al., 2017). In the first phase, we crowdsourced the development of the items to the participants, to design the items on barriers and support mechanisms, with consideration of the literature. Crowdsourced information is professional information provided by non-professionals, where the participants are the creators of the content. Crowdsourcing research is a dynamic process of data collection and analysis, where the researchers conduct a more in-depth analysis of the research objects. The participants were sophomores from an application-oriented university in Guangdong Province, China. Application-oriented universities are oriented to serve economic and social development needs and highlight the education and practice of youth in innovation and entrepreneurship. The universities’ main purpose is to cultivate more innovative, inter-disciplinary, and practical talents to contribute to local high-quality development.

In the second stage, based on the items established in the first stage, a questionnaire survey method was used to survey young people studying in Guangdong Province. Guangdong is the largest provincial economy in China and one of the provinces with the most unbalanced regional development in terms of economy and education. Two first-tier cities located in the province, namely Guangzhou and Shenzhen, which have a population of more than 16 million, attract many youths, resulting in the outflow of young people from rural areas. With the implementation of the *Outline Development Plan for the Guangdong-Hong Kong-Macao Greater Bay Area*, the Greater Bay Area has advantages in terms of the economy, industry, employment, and talent attraction due to policy support. This also aggravates the outflow of young talent from rural areas.

## Research I: Identification of the items on “barriers and support mechanisms”

### Participants, design, and procedure

In the first stage, all respondents participated in a 32-h innovation and entrepreneurship course that was compulsory, among which 16-h were conducted online. They learned the basics and theories of entrepreneurship. In the 16-h offline course, students worked in groups to make entrepreneurial proposals. The student teams observed the needs of the market and proposed solutions based on their imagination and design. During the fourth and seventh weeks, the teams used the elevator pitch approach to perform two rounds of competitive briefings. They had 1–3 min each time to quickly and succinctly communicate the key differentiating elements of their business philosophy. Finally, the students were encouraged to participate in the College Students’ Innovative Entrepreneurial Training Plan; China International College Students’ “Internet Plus” Innovation and Entrepreneurship Competition; and the National College Students E-commerce Challenge on Innovation, Creativity, and Entrepreneurship with their initial results. Rural entrepreneurship is one of the key themes of these competitions.

In research, crowdsourcing is used to generate ideas, collect data, and analyze large volumes of unstructured information (Edgar et al., 2016). In recent years, crowdsourcing has been applied to the survey process, including item development (Wang et al., 2017), market prediction (Lang et al., 2016) and creative processes (Piyathasanan et al., 2018). This study collected the personal information and opinions of the participants, mainly asking them about their opinions regarding their implementation of the entrepreneurial plans in their hometowns. The major questions in the questionnaire included (1) What do you think about returning to your hometown to start a business? (2) What are your current shortcomings? (3) What training or assistance do you need?

At the end of the course, we distributed online questionnaires through the course platform to gather the personal information and opinions of the participants. Ethical review and approval were not required for the study on human participants in accordance with the local legislation and institutional requirements. Written informed consent for participation was also not required for this study in accordance with the national legislation and institutional requirements. A total of 3,897 sophomores received the questionnaires and 2,174 questionnaires were returned. Six-hundred and three students provided 1,068 sets of opinions on return-home entrepreneurship and the assistance they needed. Their average age was about 22 years. Male students constituted 41.6% of the participants, and they were from nine colleges studying arts, science, engineering, and business.

### Results

Two assistants provided definitions and examples of the categories based on feedback from the participants. Based on the literature, we edited and discussed the item lists of all opinions to construct the category framework (Hsu, 2018). The barriers and support mechanisms were summarized in reference to the relevant literature that investigated the return-home situations of young people. Table 1 shows the specific classifications.

**TABLE 1:** Return-home entrepreneurship.

Item	Definition	Number of items	Example	References
**Barriers to entrepreneurship**				
Hometown factors	Hometown entrepreneurial environment	11	Insufficient infrastructure in hometown	Kouriloff (2000), Giacomin et al. (2011), Singh Sandhu et al. (2011)
Psychological factors	Personal thoughts on return-home entrepreneurship	5	Grim personal determination	
Entrepreneurial experience and ability	The ability to master a field or discover a new one	8	Lack of ability to identify market opportunities	
Team formation and operations	Amassing people with different expertise and turning them into a holistic, effective work unit	4	Difficulties in building a startup team	
Entrepreneurial environment	The circumstances and situations faced by entrepreneurs	7	Bad entrepreneurial environment	
Startup incubation	Providing a series of instructions and services for startup companies	4	Lack of organizations to assist entrepreneurship	
Family and connections	The family can provide resources to support the student’s return-home entrepreneurship	4	Lack of connections	
Personal factors	The student’s considerations for future careers	6	Wanting to become an employee first	
**Support mechanisms**				
Guidance for return-home entrepreneurship	Assistance on how to return home and start a business	6	Rural entrepreneurship courses	Fogel (2001); Giacomin et al. (2011)
Entrepreneurship course and technology development	Knowledge sharing and guidance, practices about entrepreneurship	8	Innovation and entrepreneurship courses	
Innovation and entrepreneurial ability	Helping students raise their level of innovation and entrepreneurship	5	Organizing innovation and entrepreneurship competitions	
Financial support	Support related to finance	6	Financing channel recommendation	
Team formation and operations	Assistance on how to form and run a team	8	Entrepreneurial risk management	
Startup incubation	Provide related services for entrepreneurship	4	Guidance from a professional mentor team	

## Research II: Guangdong province youth

### Participants, design, and procedure

We mainly looked for respondents among young people who were studying or had studied at universities in Guangdong Province. In terms of agriculture, Guangdong is a comprehensive agricultural area with comprehensive development. Its economy is dominated by agriculture, supplemented by forestry, animal husbandry, and fisheries, with a total output value of 621.49 billion RMB in 2017. To implement the central government’s policy for Sannong (agriculture, rural areas, and farmers), the government of Guangdong Province has, for years, actively promoted agricultural and rural reforms, and the agricultural and rural economy has shown a trend of sustained and rapid development. The local government has introduced many policies to accelerate rural revitalization so that rural entrepreneurship gets new growth opportunities. Many challenges and problems have arisen at the same time. For example, the structural problems in agriculture are still prominent, while the development of multifunctional agriculture is generally weak. Second, agricultural labor is characterized by an overall shortage and structural imbalance. Third, the level of industrial development needs to be improved urgently. Therefore, more talents are crucially needed in the construction of rural entrepreneurship.

The questionnaire consisted of four parts: entrepreneurial intention, hindering factors, support mechanisms, and personal information. Entrepreneurial intention included general entrepreneurial intention, the intention to use the resources of the hometown (such as agricultural products or intangible cultural heritage) for entrepreneurship, and the intention to return home for entrepreneurship. A five-point Likert scale was adopted in this part. Hindering factors were divided into 8 categories, including hometown, psychology, innovation, and entrepreneurial ability, with a total of 50 questions. Support mechanisms were divided into 7 categories, including entrepreneurship courses, technology development, and financial support, with a total of 37 questions. During the survey, the questions were not further classified. They were only divided into two major categories, entrepreneurship barriers and support mechanisms, and the options were ranked alphabetically. Multiple-choice questions were adopted in the section on hindering factors and support mechanisms. Participants were asked to choose the option that best described their feelings. When presenting the results, a discussion was conducted according to the categories in Table 1. Finally, personal information included the students’ home setting, gender, and age.

We adopted snowball sampling in the survey, recruiting people from college-level institutions or above and asking them to forward the digital questionnaire to college students who were studying or had graduated from institutions in Guangdong Province. When a respondent answered a question in at least 90 s, they would receive a reward of 3 RMB. After receiving the questionnaires, we obtained the valid questionnaires by screening and paid 3 RMB for each questionnaire to the one who forwarded it. A total of 1,150 questionnaires were received in this survey. According to the definition of young people’s engagement in return-home entrepreneurship, and after eliminating the questionnaires answered too quickly, there were 897 valid questionnaires, with a return rate of 78%. In order to encourage the formulation of entrepreneurship strategies according to various aspects relating to the different origins of the students, and to discover the differences, the students’ home settings were divided into an urban group and a rural group for comparative analysis.

In the sample, 49.6% of the participants belonged to the rural group, while 50.4% belonged to the urban group; The male participants accounted for 42.3% and the female participants accounted for 57.7%. The respondents were from 45 cities, and the top 5 were Zhanjiang (11.6%), Jieyang (10.4%), Yangjiang (9.1%), Shantou (8.9%), and Maoming (7.8%). They were from a total of 117 universities. The top five institutions were Guangdong University of Technology (8.8%), Guangzhou City University of Technology (3.8%), Guangdong Polytechnic of Water Resources and Electric Engineering (3.2%), Zhanjiang University of Science and Technology (3.1%), and the University of Electronic Science and Technology of China, Zhongshan Institute (2.6%).

### Entrepreneurial intention

This section shows the respective results of the *t*-tests performed targeting different categories of students’ home settings and their entrepreneurial intention. First, the study found that the general entrepreneurial intention of rural youth was significantly higher than that of urban youth (Mean_Rural_ = 3.427, Mean_urban_ = 3.215, *t* = 3.933, *P* < 0.001). Second, there were diverse forms of entrepreneurship that could help the development of the hometown, which did not necessarily require the return of the entrepreneurs. For example, entrepreneurs could sell specialty products from their hometowns in cities. The entrepreneurial intention of young people dropped significantly regardless of whether they were from rural or urban areas. There were also significant differences between different home settings of students (Mean_Rural_ = 3.191, Mean_urban_ = 3.060, *t* = 2.549, *P* < 0.05). Finally, when the respondents were asked about their intention to return home for entrepreneurship, their entrepreneurial intention decreased on average. The intention of rural youth to return home for entrepreneurship was significantly higher than that of urban youth (Mean_Rural_ = 3.117, Mean_urban_ = 3.000, *t* = 2.152, *P* < 0.05). In all scenarios, the entrepreneurial intention of rural youth was significantly higher than that of urban youth. Next, according to the categories in Table 1, we will separately illustrate the hindering factors and required support mechanisms that affect the intention to return home for entrepreneurship.

### Hindering factors

#### Factors related to home setting

The percentage of each factor is the occurrence of each item as a proportion of different home settings of students (rural and urban). Table 2 shows that insufficient facilities (58.5%), a low level of economic development (49.1%), poor geographical location (43.3%), and inconvenient transportation (40.2%) were the main hindering factors related to hometowns. In rural areas, the top five hindering factors related to hometowns were insufficient infrastructure (66.5%), a low level of economic development (55.7%), poor geographical location (51.0%), inconvenient transportation (51.0%), and a shortage of educational resources (44.7%). Respondent S45 (S refers to participants in Research II) pointed out that “My hometown is relatively remote, and the infrastructure is lagging behind.” S63 said, “The economic level is relatively low, and people pay attention to discounts rather than quality when purchasing products. so the enthusiasm for entrepreneurship is not very high.” Insufficient infrastructure (50.7%), a low level of economic development (42.5%), and poor geographical location (35.6%) were the factors that concerned urban youth the most. In addition to the factor of fierce competition, we found that in the responses of the rural youth, the percentages of various hindering factors related to their hometowns were significantly higher than in the responses of the urban youth.

**TABLE 2:** Hometown factors.

Hindering factors	Rural youth		Urban youth		All respondents	
			
	Frequency	%	Frequency	%	Frequency	%
Insufficient infrastructure	296	66.5	229	50.7	525	58.5
Low level of economic development	248	55.7	192	42.5	440	49.1
Poor geographical location	227	51.0	161	35.6	388	43.3
Inconvenient transportation	227	51.0	134	29.6	361	40.2
Shortage of educational resources	199	44.7	133	29.4	332	37.0
Unclear development prospects	176	39.6	151	33.4	327	36.5
Loss of labor force	192	43.1	134	29.6	326	36.3
Indistinct features	147	33.0	124	27.4	271	30.2
Insufficient resources	135	30.3	93	20.6	228	25.4
Unfamiliarity with the hometown	50	11.2	31	6.9	81	9.0
Fierce competition	25	5.6	46	10.2	71	7.9

#### Psychological factors

In terms of psychological factors (Table 3), the young people indicated that ability (25.3%), determination (20.0%), and confidence (19.1%) were the main factors hindering their return-home entrepreneurship. F1128 (F refers to the participants in Research I) said, “My ability is limited, and I lack the relevant knowledge and skills.” F1023 pointed out that return-home entrepreneurship was a “matter of determination.” Although rural young people were worried that their ability was limited, their percentages of choosing other items were significantly lower than those of urban youth. This indicates that the rural youth were not afraid of failure, but were worried about their own incompetence.

**TABLE 3:** Psychological factors.

Hindering factors	Rural youth		Urban youth		All respondents	
			
	Frequency	%	Frequency	%	Frequency	%
Limited ability	115	25.8	112	24.8	227	25.3
Grim determination	76	17.1	103	22.8	179	20.0
Lack of confidence	75	16.9	96	21.2	171	19.1
Fear of failure	43	9.7	67	14.8	110	12.3
Lack of perseverance	44	9.9	63	13.9	107	11.9

#### Entrepreneurial experience and ability

Table 4 shows that the shortage of entrepreneurial ability came from a lack of entrepreneurial experience (41.9%), poor ability to identify market opportunities (32.6%), and a lack of understanding of the business world and the market (32.4%). For the rural youth, the top three factors were the lack of entrepreneurial experience (40.7%), lack of ability to identify market opportunities (33.3%), and lack of understanding of the business world and the market (32.8%). The urban youth indicated that what they lacked the most were entrepreneurial experience (43.1%), understanding of the business world and the market (32.1%), and the ability to identify market opportunities (31.9%). F1451 stated, “I don’t have enough entrepreneurial experience.” F777 said, “University students do not understand the real entrepreneurial market, are inexperienced, and are prone to failure.” Therefore, young people in Guangdong Province seemed to be slightly lacking in experience, the ability to identify markets, and understanding of the business world and markets. According to the 2019 Chinese College Students Entrepreneurship Report released by Renmin University of China (2020), university students believe that there are shortfalls in entrepreneurship courses and practical activities. Therefore, one of the factors that limits the development of youth entrepreneurship in China nowadays is the lack of effective ways for them to gain entrepreneurial experience.

**TABLE 4:** Entrepreneurial experience and ability.

Hindering factors	Rural youth		Urban youth		All respondents	
			
	Frequency	%	Frequency	%	Frequency	%
Lack of entrepreneurial experience	181	40.7	195	43.1	376	41.9
Lack of ability to identify market opportunities	148	33.3	144	31.9	292	32.6
Lack of understanding of the business world and the market	146	32.8	145	32.1	291	32.4
Lack of entrepreneurial ideas or plans	121	27.2	137	30.3	258	28.8
Lack of professional knowledge	112	25.2	113	25.0	225	25.1
Lack of keen business acumen	115	25.8	108	23.9	223	24.9
Lack of technology	97	21.8	87	19.2	184	20.5
Unclear entrepreneurial directions	79	17.8	100	22.1	179	20.0

#### Team formation and operations

Table 5 reveals that the lack of financial management experience (23.5%) and management experience (22.9%) were the top hindering factors among the participants. We found that there was a significantly higher proportion of rural youth encountering difficulty in building up a startup team than urban youth. Therefore, today’s young people lack financial management and team management skills, and rural youth find it difficult to form teams. F767 believed that “inexperience, without a suitable team and capital flow, may lead to an increased likelihood of failure.” Inadequate management of capital is one of the major problems that hinders university students’ entrepreneurship. Even if they undertake a good business project in the startup stage, the company is likely to face liquidity crunches in its operations, making it impossible for the startup to succeed. Poor financial literacy and inappropriate management practices limit entrepreneurial activities (Kojo Oseifuah, 2010). Furthermore, F1468 pointed out, “I do not know much about my hometown, and I cannot find like-minded people.” This may be because it is more difficult for young people from rural areas to find like-minded people and return to their hometowns to start a business together. Compared with starting a business alone, starting a business in a team provides more resources, a wider diversity of viewpoints, greater risk tolerance, and a broader range of ideas (Barringer et al., 2005).

**TABLE 5:** Team formation and operations.

Hindering factors	Rural youth		Urban youth		All respondents	
			
	Frequency	%	Frequency	%	Frequency	%
Lack of financial management experience	103	23.1	108	23.9	211	23.5
Lack of management experience	102	22.9	103	22.8	205	22.9
Difficulties in building a startup team	94	21.1	76	16.8	170	19.0
Inability to find like-minded people	73	16.4	80	17.7	153	17.1

#### Entrepreneurial environment

The analysis of the entrepreneurial environment is shown in Table 6. Excessive risk (20.8%), difficulties in entrepreneurship (19.8%), and few entrepreneurial opportunities (19.0%) were found to be the main environmental factors hindering youth from returning to their hometowns. S1230 pointed out that return-home entrepreneurship is “very meaningful, but the entrepreneurial risks are also high. It needs to be considered in many ways and implemented carefully with comprehensive consideration of various factors.” S1494 believed that his/her hometown “has few resources and it will be difficult to start a business there.” The rural youth’s percentages of agreeing with the factors of few entrepreneurial opportunities, bad entrepreneurial environment, and excessive risk were higher than those of urban youth by 6.5, 4.0, and 3.2%, respectively. This indicates that young people from rural areas believe that there are more opportunities and greater development potential in urban areas, while there are few opportunities and an unfavorable entrepreneurial environment in rural areas, posing more risks and difficulties. S752 pointed out, “This [return-home entrepreneurship] can increase employment, but to be honest, there are greater development potentials and more opportunities in big cities.” However, some participants did not agree. S144 stated, “My hometown is in a period of rapid development, and there are more entrepreneurial opportunities. If I have the chance to start a business in my hometown and support its development, I can benefit other people as well as myself and can develop the specialties of my hometown.” This further indicates that although entrepreneurship comes with risk, where there is risk, there is opportunity. Many aspects should be considered before a university student chooses to start a business, and this cannot be done overnight. If they find a suitable opportunity to start a business in their hometown, they may choose return-home entrepreneurship.

**TABLE 6:** Entrepreneurial environment.

Hindering factors	Rural youth		Urban youth		All respondents	
			
	Frequency	%	Frequency	%	Frequency	%
Excessive risks	100	22.5	87	19.2	187	20.8
Difficulties in entrepreneurship	93	20.9	85	18.8	178	19.8
Few entrepreneurial opportunities	99	22.2	71	15.7	170	19.0
Bad entrepreneurial environment	81	18.2	64	14.2	145	16.2
Current economic situation	61	13.7	52	11.5	113	12.6
Excessive labor wages	28	6.3	36	8.0	64	7.1
Startup paperwork and bureaucracy	33	7.4	30	6.6	63	7.0

#### Startup incubation

As can be seen from Table 7, the main barriers to startup incubation were the lack of policy assistance or consultation (19.4%) and the lack of formal help to start a business (15.8%). F779 emphasized “the analysis of current policies and how the policies are concretized.” Support mechanisms or incentive systems can encourage people to be more sensitive to potential profit opportunities and to act upon value propositions. Studies have shown that the more people know about a policy, the higher their satisfaction with the policy, and this is especially true in developing countries (Minniti, 2008; Xiong et al., 2018; Morris and Tucker, 2021). In addition, F1383 pointed out, “I am inexperienced and do not know much about entrepreneurial matters.” F1320 said, “I believe that everything is possible, but the theoretical knowledge that I have is limited. If I decide to return to my hometown to start a business after graduation, I need to spend time gaining theoretical knowledge.” This shows that students in China are used to acquiring knowledge before taking action. Rural students indicated that they mostly lacked policy assistance or consultation (21.3%) and formal help to start a business (16.9%). On the other hand, the urban students indicated that they lacked policy assistance or consultation (17.5%) and assistance in assessing business viability (15.0%). Many respondents said they need policy support from the local government, which is considerably helpful to young people engaging in return-home entrepreneurship and can attract them to do so.

**TABLE 7:** Startup incubation.

Hindering factors	Rural youth		Urban youth		All respondents	
			
	Frequency	%	Frequency	%	Frequency	%
Lack of policy assistance or consultation	95	21.3	79	17.5	174	19.4
Lack of formal help to start a business	75	16.9	67	14.8	142	15.8
Lack of assistance in assessing business viability	66	14.8	68	15.0	134	14.9
Lack of organizations to assist entrepreneurship	71	16.0	57	12.6	128	14.3

#### Family and connections

Table 8 shows that the lack of *guanxi* (32.6%) and lack of family support due to weak financial conditions (20.4%) are the hindering factors that concern the youth the most. Whether in rural or urban areas, *guanxi* and family factors have a great influence on youth entrepreneurship. *Guanxi* refers to interpersonal connections and can bring a wide range of benefits in Chinese communities (Fan, 2002). In the early stage of entrepreneurship, an entrepreneur mainly relies on personal networks to obtain entrepreneurial information and business opportunities. Participant F1401 stated, “I don’t have many *guanxi* connections [in my hometown] as I am a student. Students from other cities are all the connections that I have.” Young people need to expand their social networks in their hometowns and enrich the diversity of the relationships in their networks in order to obtain the key information and resources they need from these networks. Respondent S696 said, “Starting a business is risky, and for most of us students, we are not willing to take the risk if we are not from a wealthy family. The thing we want is simply getting a stable job.” The risk of starting a business will affect the economic interests of the entire family. Many university students refuse to let their families take all the risks and choose to look for a stable job.

**TABLE 8:** Family and connections.

Hindering factors	Rural youth		Urban youth		All respondents	
			
	Frequency	%	Frequency	%	Frequency	%
Lack of guanxi connections	142	31.9	150	33.2	292	32.6
Lack of family support due to weak financial condition	97	21.8	86	19.0	183	20.4
Lack of support from those around me (such as family and friends)	55	12.4	39	8.6	94	10.5
Parents have left the hometown	29	6.5	15	3.3	44	4.9

#### Personal factors

It can be seen from Table 9 that the lack of startup capital (39.8%) is the greatest hindering factor, followed by wanting to become an employee first (17.1%) and wanting to develop a career away from the hometown first (10.9%). First, a lack of capital was the most frequently mentioned problem, and most young people believed that starting a business requires a lot of capital. S811 pointed out that “more assistance can be given to young entrepreneurs to solve the financing difficulties of small enterprises.” Young entrepreneurs usually get their startup capital from their families, which was confirmed by the participants (Table 8). Compared with other sources of financial support, young people will feel more at ease and more encouraged if they are supported with funds from their families, and this is suitable for the initial process of entrepreneurship (Pérez-Macías et al., 2019). Secondly, S1464 stated that “At the moment, I cannot find any [startup] project that can be implemented in the hometown, and I develop my career mainly in the city.” F1129 also stated, “My hometown is developing gradually, but the growth speed and current situation still lag far behind the city in which I live. Therefore, I don’t think there is a good prospect for starting a business back home.” However, respondent S1064 pointed out, “If I have a higher level of competencies and financial resources in the future, I will choose to return to Huizhou to make my own contribution to the city.” S453 explained, “My hometown is, indeed, to be developed. If I have the opportunity or the competencies, I may contribute to the education, tourism resources, or service industry in my hometown.” It can be seen that personal factors also significantly affect the return-home entrepreneurship decisions of university students.

**TABLE 9:** Personal factors.

Hindering factors	Rural youth		Urban youth		All respondents	
			
	Frequency	%	Frequency	%	Frequency	%
Lack of startup capital	177	39.8	180	39.8	357	39.8
Wanting to become an employee first	74	16.6	79	17.5	153	17.1
Wanting to develop a career away from the hometown first	45	10.1	53	11.7	98	10.9
Life planning	29	6.5	31	6.9	60	6.7
Having no interest	17	3.8	29	6.4	46	5.1
Excessive working hours	16	3.6	17	3.8	33	3.7

#### Summary of barriers to entrepreneurship

The top five factors hindering rural young people’s engagement in return-home entrepreneurship were insufficient infrastructure (66.5%), a low level of economic development (55.7%), poor geographical location (51.0%), inconvenient transportation (51.0%), and a shortage of educational resources (44.7%). For urban youth, the top five hindering factors were insufficient infrastructure (50.7%), a lack of entrepreneurial experience (43.1%), a low level of economic development (42.5%), a lack of startup capital (39.8%), and poor geographical location (35.6%). The factors that hindered the rural youth’s return-home entrepreneurship were all related to their hometowns, while for urban youth they were mostly related to experience and funding.

### Support mechanisms

#### Guidance on development in hometown

Table 10 shows young people’s views on the guiding mechanism for return-home entrepreneurship. All respondents mostly valued guidance on return-home entrepreneurship (44.5%), courses on hometown development status (29.9%), courses on rural entrepreneurship (28.4%), and establishing cooperation with hometown enterprises (26.2%). Compared with urban youth, we found that rural youth have a strong demand for information about their hometown. F592 said, “I hope the school can offer training courses on return-home entrepreneurship and teach us where to start.” F1252 stated, “The support from the environment in the hometown is necessary for return-home entrepreneurship. If there is no such support [entrepreneurship environment, legal order, and local policies], it is impossible for us to do anything.” This implies that after gaining theoretical knowledge in school, it is necessary for young people to fully understand the development status of their hometowns before they start a business there. There is no shortage of entrepreneurial resources in rural areas, but for return-home entrepreneurship, young people need assistance in combining the use of resources, theoretical knowledge, and entrepreneurial ideas. Universities should conduct more relevant activities to encourage university students to launch village and hometown enterprises.

**TABLE 10:** Guidance on return-home entrepreneurship.

Facilitating factors	Rural youth		Urban youth		All respondents	
			
	Frequency	%	Frequency	%	Frequency	%
Guidance on return-home entrepreneurship	209	47.0	190	42.0	399	44.5
Courses on hometown development status	170	38.2	98	21.7	268	29.9
Courses on hometown entrepreneurship	162	36.4	93	20.6	255	28.4
Establishing cooperation with hometown enterprises	123	27.6	112	24.8	235	26.2
Return-home entrepreneurship practice	99	22.2	81	17.9	180	20.1
Entering into hometown activities	97	21.8	72	15.9	169	18.8

#### Entrepreneurship courses and technological development

Table 11 reveals that the rural and urban youth had similar views and had a high demand for knowing how to start a business. Nearly half of the participants were interested in entrepreneurial experience sharing (63.0%), innovation and entrepreneurship courses (55.34%), and cases of entrepreneurship (47.5%). Young people believed that the sharing of experiences by entrepreneurs would increase their motivation to start a business. F512 believed that there should be “courses that introduce in detail the entire entrepreneurial process, as well as the problems encountered and the solutions.” In China, entrepreneurial experience providers mostly provide know-what experience and passively transfer knowledge through classroom teaching, seldom explaining the know-how (Zheng et al., 2017). Students have an urgent need for information on entrepreneurial practices, such as case studies and business startup sharing. F1955 suggested “offering various entrepreneurship courses.” Since 2012, the Ministry of Education of China has required universities to offer a two-credit compulsory course called “Foundation of Entrepreneurship” to all undergraduate students, and stipulates that the course be included in education and teaching quality evaluation indicators. However, according to our survey, there is still a great demand for entrepreneurship-related courses among students. Universities should reorganize the traditional educational programs and approaches to create an environment that enables students to start a business and supports them through the process from the birth of an idea to the development of the idea, the production of a prototype, and commercialization (Secundo et al., 2021).

**TABLE 11:** Entrepreneurship courses and technology development.

Facilitating factors	Rural youth		Urban youth		All respondents	
			
	Frequency	%	Frequency	%	Frequency	%
Entrepreneurial experience sharing	280	62.9	285	63.1	565	63.0
Innovation and Entrepreneurship courses	252	56.6	244	54.0	496	55.3
Technological guidance	220	49.4	220	48.7	440	49.1
Cases of entrepreneurship	211	47.4	215	47.6	426	47.5
Entrepreneurship seminars	172	38.7	185	40.9	357	39.8
Practical courses	131	29.4	142	31.4	273	30.4
Technology development course	132	29.7	123	27.2	255	28.4
Co-creation by teachers and students	85	19.1	79	17.5	164	18.3

Technological innovation is the main engine that drives the rapid growth of agriculture, and the resources allocated for technology are the key to improving the level of technological innovation for agriculture. Table 11 shows that technology was slightly more important to rural youth than to urban youth when deciding whether to return home and start a business. Participant F554 suggested “providing guidance on technology and knowledge,” while S31 believed that there is a “lack of financial and technological support.” In the agricultural sector, some farmers have lower productivity, and most of them are smallholder farmers and elderly people. As a country with huge natural resources, China’s agricultural sector is a business sector with a promising future that needs the input of young people. The development of agricultural entrepreneurship is even more essential to increase the human resource productivity in the sector. High-quality science and technology are needed to promote the development of modern agriculture and improve the productivity and economic benefits of agriculture. Therefore, in the process of cultivating innovative talents with expertise in agriculture, education managers must focus on the cultivation of students’ scientific and technological abilities.

#### Cultivation of innovation and entrepreneurial ability

According to Table 12, the proportions of innovative thinking (28.7%), entrepreneurial ability (27.0%), and interdisciplinary learning (25.9%) were all more than a quarter. Rural youth valued innovative thinking (29.9%) and entrepreneurial ability (28.1%), while urban youth focused on innovative thinking (27.4%) and interdisciplinary learning (26.3%). Innovative thinking is conceptualized as generating creative ideas (Geldhof et al., 2014), and it was valued by these two groups of young people. This will allow them to propose innovative solutions as they discover new business opportunities, so as to better fulfill the needs of consumers. Entrepreneurial ability is a crucial quality for entrepreneurs. If they start a business blindly without having sufficient ability, their entrepreneurship endeavors are prone to fail. In urban areas, as the competition is fierce, training in interdisciplinary and comprehensive skills can effectively improve students’ innovation and entrepreneurial ability. F805 said, “The practical courses in innovation and entrepreneurship expand our realm of imagination and thinking, and are a way for us to understand entrepreneurial knowledge.”

**TABLE 12:** Cultivation of innovation and entrepreneurial ability.

Facilitating factors	Rural youth		Urban youth		All respondents	
			
	Frequency	%	Frequency	%	Frequency	%
Innovative thinking	133	29.9	124	27.4	257	28.7
Entrepreneurial ability	125	28.1	117	25.9	242	27.0
Interdisciplinary learning	113	25.4	119	26.3	232	25.9
Self-confidence building	113	25.4	100	22.1	213	23.7
Organizing innovation and entrepreneurship competitions	80	18.0	71	15.7	151	16.8

The demand for building self-confidence among rural youth was 3.3% higher than among urban youth. In the entrepreneurial process, self-confidence can help young people reduce their fear and anxiety, gain greater motivation, and be more resilient to setbacks, which is extremely important in the process of starting a business. Interestingly, less than 20% of young people showed a demand for innovation and entrepreneurship competitions. Since 2016, innovation and entrepreneurship courses have become compulsory for Chinese university students, and students are encouraged to participate in entrepreneurship competitions. Promoting innovation and entrepreneurship through competitions has become a Chinese model of innovation and entrepreneurship education.

#### Financial support

The respondents’ views on financial support are shown in Table 13. For all respondents, the proportion of startup capital was 47.5%, followed by financial support (46.2%) and incentive measures (33.6%). A comparison of the data of rural youth and urban youth showed that rural youth had a higher demand for funds than urban youth. In particular, in terms of startup capital, financial support, incentive measures, and funds to encourage return-home entrepreneurship, the levels of concern of rural youth were higher than those of urban youth by 2–5%. Among them, the most obvious differences were in financial support and funds to encourage return-home entrepreneurship, in which the levels of concern of rural youth were higher than those of urban youth by nearly 5.6%. The lack of effective financing channels was the main problem in the development process of young people’s engagement in return-home entrepreneurship projects. Agriculture-related industries usually adopt an asset-heavy investment mode and need the support of a large amount of funding. The government should increase the support for young rural entrepreneurs and solve the financing difficulties of small and micro-enterprises.

**TABLE 13:** Financial support.

Facilitating factors	Rural youth		Urban youth		All respondents	
			
	Frequency	%	Frequency	%	Frequency	%
Startup capital	217	48.8	209	46.2	426	47.5
Financial support	218	49.0	196	43.4	414	46.2
Incentive measures	160	36.0	141	31.2	301	33.6
Funds to encourage return-home entrepreneurship	149	33.5	126	27.9	275	30.7
Financing channel recommendation	123	27.6	115	25.4	238	26.5
Studio rent	106	23.8	106	23.5	212	23.6

#### Team formation and operations

Table 14 presents the data on team formation and operations. In each of the items, the proportion was nearly 30% above, showing that there is a high demand for the items in this category among young people. Training in regulations (46.7%), talent cultivation (41.5%), and market analysis (41.4%) were rated as the most important items. Li et al. (2020) pointed out that China issued as many as 66,382 laws, regulations, and policies on “entrepreneurship” or the “promotion of employment” between 2000 and 2016. From the perspective of young people, it is not easy to completely understand the overall situation and evolution of China’s entrepreneurship policy from a macro perspective. Traditional programs for the cultivation of university students’ innovative abilities are usually limited to a single discipline, so that the knowledge, technology, and tools they use cannot transcend the barriers between professions. Respondent S615 said, “A high-quality team is the foundation of entrepreneurship.” The shortage of talent has always been a bottleneck that affects and constrains community development. There are two types of talent cultivation. The first is to cultivate technological and technical talents that serve the country’s strategic development needs, and the second is to cultivate technological and technical talents that serve regional industries and take root locally.

**TABLE 14:** Team formation and operations.

Facilitating factors	Rural youth		Urban youth		All respondents	
			
	Frequency	%	Frequency	%	Frequency	%
Training on regulations for entrepreneurs	212	47.6	207	45.8	419	46.7
Talent cultivation	195	43.8	177	39.2	372	41.5
Market analysis	180	40.4	191	42.3	371	41.4
Operation and management course	157	35.3	152	33.6	309	34.4
Entrepreneurial risk management	146	32.8	130	28.8	276	30.8
How to start a business	137	30.8	134	29.6	271	30.2
Policy interpretation	133	29.9	131	29.0	264	29.4
Building a startup team	137	30.8	118	26.1	255	28.4

The needs of rural youth in building a startup team, talent cultivation, and entrepreneurial risk management were higher than those of urban youth. S128 pointed out that “Teamwork is necessary for entrepreneurship.” Fewer young people choose return-home entrepreneurship, and the chances of forming a return-home entrepreneurship team are even lower. The attractiveness of rural areas to talent, especially young people, is obviously low, and this has become a major barrier to young people’s engagement in return-home entrepreneurship. At the same time, rural youth have a high demand for assistance in entrepreneurial risk management. On the one hand, they worry that they cannot withstand the financial pressure brought on by the high entrepreneurial risks. On the other hand, this indicates that the experience in entrepreneurial risk management that can be gained by university students from rural areas is limited.

#### Startup incubation

The analysis of startup incubation is shown in Table 15. The major demands of young people for incubation mechanisms were pre-startup mentoring (52.1%), guidance from a professional mentor team (40.0%), and consultation (31.8%). F1713 said, “I [need] guidance on the entrepreneurial team and about the early stage of starting up a business.” Entrepreneurial mentoring is an intervention usually aimed at accelerating the growth of startups and nascent entrepreneurs (Baluku et al., 2019). Entrepreneurial mentors’ guidance to entrepreneurs is one of the effective ways to sharpen their entrepreneurial skills. The needs for startup incubation among rural and urban youth are similar. When potential entrepreneurs come up with an idea, they begin to collect information continuously to make the idea clearer. In the early stage of a startup business, rural entrepreneurs usually need to revise their entrepreneurial ideas through high trust and close communication. If they have small personal networks, entrepreneurs will look for incubation platforms. F134 said, “[I hope that] the startup incubation bases can hold more gatherings relating to innovation and entrepreneurship knowledge, so that students can better understand entrepreneurship.” Outsider consultation and assistance have a greater impact on the starting up, survival, and performance of new businesses compared to those provided by schools (Chrisman and McMullan, 2004).

**TABLE 15:** Startup incubation.

Facilitating factors	Rural youth		Urban youth		All respondents	
			
	Frequency	%	Frequency	%	Frequency	%
Pre-startup mentoring	235	52.8	232	51.3	467	52.1
Guidance from a professional mentor team	181	40.7	178	39.4	359	40.0
Consultation	134	30.1	151	33.4	285	31.8
Innovation and entrepreneurship platform	126	28.3	119	26.3	245	27.3

#### Summary of support mechanisms

Rural youth had a high demand for entrepreneurial experience sharing (62.9%), innovation and entrepreneurship courses (56.6%), pre-startup mentoring (52.8%), technological guidance (49.4%), and financial support (49.0%). Urban youth valued entrepreneurship experience sharing (63.1%), innovation and entrepreneurship courses (54.0%), pre-startup mentoring (51.3%), technological guidance (48.7%), and cases of entrepreneurship (47.6%). The support mechanisms needed by the two groups were similar; that is, there was a high demand for entrepreneurial experience sharing. Entrepreneurial experience refers to the perceptual or intellectual concepts, knowledge, and skills obtained by entrepreneurs from the experience of starting a business. Young people can gain entrepreneurial confidence through the sharing of entrepreneurs, which helps them believe that they have the ability to solve the problems they may face during the later stages of starting a business.

## Discussion

There has been a wave of return-home entrepreneurship in China in recent years, which is essentially the return migration of more and more peasant workers from cities to their rural homes, who choose to become self-employed afterward. The education level of the majority of return-home entrepreneurs is junior high school or below. As the rural youth study and work in big cities, they acquire technical expertise, as well as management and entrepreneurial skills. Highly educated youth enjoy systemic advantages in perceiving opportunities, developing entrepreneurial intentions or behaviors, and undertaking entrepreneurial activities (Minola et al., 2014). In the context of rural revitalization, China’s Ministry of Education encourages young people to focus on rural areas by holding entrepreneurship competitions. Young people’s engagement in return-home entrepreneurship is affected by multiple factors, and the complexity of the influencing factors brings about daunting challenges in the development of return-home entrepreneurship. To assist the youth who engage in return-home entrepreneurship or have the intention to do so, it is most important to identify which key factors and support mechanisms should be used to determine the directions of policies to ensure the rapid development of return-home entrepreneurship.

Through a two-stage approach, this study collected a huge amount of data to examine the opinions of youth on return-home entrepreneurship. The results of this study are summarized as follows: (1) Compared with general entrepreneurship, the intention to return home for entrepreneurship has declined to a certain extent. In rural areas, young people consider lagging environmental development to be the biggest barrier. In urban areas, infrastructure, a lack of entrepreneurial experience, and funding are the factors that attract the most attention from young people. (2) As they have limited entrepreneurial experience, young people have a high demand for entrepreneurial experience sharing, as well as entrepreneurship courses and mentoring. (3) The startup capital needed for return-home entrepreneurship is a common problem faced by young people. (4) Young people are not afraid of failure, but they are worried about their incompetence in entrepreneurship, including the lack of the ability to explore market opportunities and propose entrepreneurial ideas. (5) In terms of the entrepreneurial environment, rural areas still lag behind urban ones. (6) Rural youth’s need for startup incubation is significantly higher than that of urban youth.

### Theoretical and practical implications

Rural youth believe that the infrastructure in rural areas is inferior to that in urban environments. Appropriate infrastructure should be developed to support their entrepreneurship. Rural entrepreneurship faces many barriers, including insufficient resources (human, financial, and knowledge), unusable infrastructure, and poor market access (Abeysinghe and Malik, 2021). This is consistent with the results of previous studies, which discovered that many developing countries have similar problems. For example, infrastructure is one of the main factors hindering the entrepreneurial development of Iran’s agricultural production cooperatives (Azari et al., 2017). The sticky problem of infrastructure and the absence of a system to support market economies may negatively affect public attitudes toward entrepreneurship in developing economies (Ozgen and Minsky, 2007). However, this study showed that although cultural differences between urban and rural areas, the economic levels of rural areas, and other factors restrict the return-home entrepreneurship of the youth, they also have advantages related to policies and geography. Against the backdrop of the rural revitalization strategy, as long as the number of people engaging in return-home entrepreneurship increases, the entrepreneurial environment will improve.

University students are young, inexperienced, and may be reluctant to take risks. Business experience has a significant impact on young people’s entrepreneurial intention (Wang and Wong, 2004). Therefore, to reduce the chance of failure when starting a business, young people are eager to learn from entrepreneurs. Entrepreneurs’ experience can be divided into categories such as management experience, industry experience, and entrepreneurial experience (Morris et al., 2012). In this way, young people can understand how to deal with possible problems in the entrepreneurial journey. In terms of entrepreneurial mentoring, South Korea provides one-to-one mentoring services for those who move to rural areas and engage in agriculture or agricultural entrepreneurship (Wang et al., 2021). China has also established a mentoring system to help those returning to their hometowns make startup plans and implement them smoothly. However, this is inconsistent with the views of Mueller and Anderson (2014). They believe that the learning process of students, which is not based on entrepreneurial practice, is different from that of entrepreneurs. They suggested allowing students to discover their own shortcomings based on practice before guiding them or providing them with assistance, thereby solving their problems.

For young people, it is not easy to obtain startup capital, and this is one of the biggest barriers on the road to entrepreneurship. In this survey, nearly 40% of the respondents stated that they lacked startup capital for entrepreneurship. In the category of financial support and assistance, approximately half of the respondents were concerned about this issue, putting it in the top 10. When raising initial resources for the startup, entrepreneurs mainly rely on personal networks and obtain the needed resources by relying on informal relationships based on emotional connections, such as with relatives and friends. Most business relationships in Asia rely heavily on social networks, but this study found that as many as 30% of the respondents lacked *guanxi* connections. This is consistent with the research results of Muñoz and Kimmitt (2019), which showed that networks and social capital are regarded as the key elements in entrepreneurship, affecting how an individual can obtain services and resources in a rural environment. Furthermore, the lack of correct financing mechanisms, targeted policy formulation, and government support for rural entrepreneurship is the main factor hindering the application of the value chain’s circular development in the context of rural entrepreneurship. Governments and universities should understand how to find and nurture potential entrepreneurs, even if they are still students (Singh Sandhu et al., 2011). Efforts to improve the education, infrastructure, legal conditions, and financial support for potential entrepreneurs should be intensified.

In addition, the current talent training mechanism is not well-rounded; therefore, young people lack relevant entrepreneurial skills such as entrepreneurial and management capabilities. It is suggested to develop rural youth’s abilities in entrepreneurial planning and market analysis to enhance their strategic competence (Ataei et al., 2020). This study agrees with the views of Papagiannis (2018), who holds that removing the barriers of “capital, experience and access to professional knowledge” is the way to solve the youth’s problems in seeking access to information, and one especially useful method is holding entrepreneurship seminars. Based on the results of this study, a multi-pronged approach can be used to improve the competencies of the youth. For example, universities can conduct regular entrepreneurship seminars for their students and invite successful entrepreneurs to impart their entrepreneurial experience, conduct in-depth training in professional skills related to entrepreneurship, and provide targeted training to outstanding talents with interest and potential in entrepreneurship and the intention to return home for entrepreneurship.

The challenge for rural areas is to stimulate some would-be entrepreneurs to participate in entrepreneurial activities and to support existing entrepreneurs. The government should strengthen the implementation of education consultation policies by holding formal and informal training courses and establishing consultation centers and entrepreneurship websites that support small and medium enterprises (Ataei et al., 2020). What return-home entrepreneurs need most is the incubation support provided in industrial parks. Miles and Morrison (2020) pointed out that entrepreneurial and business skills training programs, startup training camps, startup accelerators, and business incubators are all conducive to increasing the rate of entrepreneurial activity and efficiency within a community.

### Limitations of the study and suggestions for future research

For Chinese young people to engage in return-home entrepreneurship, the existing problems of information asymmetry, poor circulation of social resources, and inadequate implementation of policies, a situation that is not favorable to them, should be addressed. The respondents in our survey were all young people studying in Guangdong Province. In the future, it is suggested to explore the barriers and support mechanisms of young people from colleges and universities in different provinces in the context of return-home entrepreneurship. There are significant differences in development between different countries or between provinces in China. It is possible to identify the barriers and support mechanisms of young people in different regions and put forward suggestions for rural development that represents a greater range of situations. Second, multiple-choice questions were used in this study, and participants were asked to choose the options according to their feelings. Future research could build on this study by using a Likert scale. Finally, there is a need for more research on the intentions of young people. It is suggested to provide further insights and information on the issues facing young people in developing countries, to reduce the differences (Singh Sandhu et al., 2011).

## Conclusion

Under background of the rural revitalization strategy, the Chinese government has introduced several policies to encourage return-home entrepreneurship among young people. In addition to solving their own employment issues, their new ventures at home can also create more jobs. Although there is a trend of return-home entrepreneurship, the education level of the majority of return-home entrepreneurs is junior high school or below. Due to the major differences in development between urban and rural areas, well-educated youth have a low willingness to return home for entrepreneurship. This study explored this issue through a combination of qualitative and quantitative methods, and found that rural youth had a stronger intention to start a business than their urban counterparts, and had different views on the barriers to entrepreneurship. In rural villages, the youth believed that the unfavorable environment for entrepreneurship and consumption, as well as the lagging development, are the biggest barriers. In urban areas, infrastructure and a lack of entrepreneurial experience and funding were the factors that concerned young people the most. As they have limited entrepreneurial experience, young people in both rural and urban areas have a high demand for information on entrepreneurial practices, including the sharing of entrepreneurial experience, as well as entrepreneurship courses and mentoring.

## Data availability statement

The original contributions presented in this study are included in the article/supplementary material, further inquiries can be directed to the corresponding author.

## Ethics statement

Ethical review and approval was not required for the study on human participants in accordance with the local legislation and institutional requirements. Written informed consent for participation was not required for this study in accordance with the national legislation and the institutional requirements.

## Author contributions

C-HY: conceptualization. DW and YZ: writing. LH: review. JW: editing. All authors contributed to the article and approved the submitted version.
